# Effectiveness of compensating filters in the presence of tissue inhomogeneities

**DOI:** 10.1120/jacmp.v4i3.2517

**Published:** 2003-06-01

**Authors:** A. Sethi, L. Leybovich, N. Dogan, G. Glasgow

**Affiliations:** ^1^ Department of Radiation Oncology Loyola University Medical Center Maywood Illinois 60153

**Keywords:** compensating filters, tissue inhomogeneity, dose uniformity

## Abstract

CT based 3D treatment planning systems (3DTPS) can be used to design compensating filters that, in addition to missing tissue compensation, can account for tissue inhomogeneities. The use of computer‐driven systems provides a practical, convenient, and accurate method of fabricating compensating filters. In this work, we have evaluated a commercially available PAR Scientific DIGIMILL milling machine linked with FOCUS 3DTPS. Compensating filters were fabricated using refined gypsum material with no additives. Thus, filters were of manageable dimensions and were not sensitive to common machining errors. Compensating filters were evaluated using a homogeneous step phantom and step phantoms containing various internal inhomogeneities (air, cork, and bone). The accuracy of two planning algorithms used to design filters was experimentally evaluated. The superposition algorithm was found to produce better agreement with measurements than the Clarkson algorithm. Phantom measurements have demonstrated that compensating filters were able to produce a uniform dose distribution along the compensation plane in the presence of tissue inhomogeneity. However, the dose variation was greatly amplified in planes located beyond the inhomogeneity when a single compensated beam was used. The use of parallel‐opposed compensated beams eliminated this problem. Both lateral and depth‐dose uniformity was achieved throughout the target volume.

PACS number(s): 87.53.–j, 87.66.–a

## INTRODUCTION

Three‐dimensional compensating filters are often used to provide corrections for missing tissue and/or tissue inhomogeneities encountered by a photon beam.^1–^, ^6^ However, with a compensating filter, a uniform dose distribution can be achieved in only a selected compensation plane (CP), which is usually positioned at the target center. In planes located above and below the CP, the filter overcompensates and undercompensates respectively, resulting in nonuniform dose distribution in these planes. The degree of dose nonuniformity increases with the increasing distance from the CP. When a photon beam passes through an inhomogeneous medium, the dose nonuniformity is significantly increased.

The automated systems, which consist of a 3D treatment planning system (3DTPS) linked with a compensator‐milling machine, have considerably increased the use of compensating filters. Compared to the older manual methods^7,^, ^8^ of designing filters, computer‐driven systems^9–^, ^13^ provide improved compensation for surface irregularities (better precision and continuous versus discrete shape profile). In addition, the CT based filter design allows for the compensation of tissue inhomogeneities.

We have evaluated compensating filters produced by a system consisting of PAR Scientific DIGIMILL (S&S Par Scientific, Inc., Brooklyn, NY) and FOCUS 3DTPS (Computerized Medical Systems, Inc., St. Louis, MO). Filters were designed to compensate for surface irregularities and various internal inhomogeneities (air, cork, and bone) for a range of photon energies. Both Clarkson^14^ and superposition^15^ algorithms were tested in designing compensating filters and evaluating the resultant dose distribution. All filters were fabricated using refined gypsum material^16–^, ^18^ without any additives.

## METHODS AND MATERIALS

**Figure 1:**
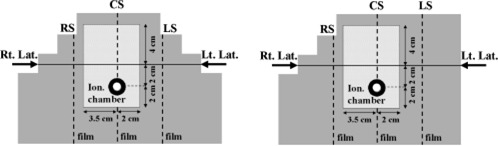
(Color) Cross sectional view of phantoms (phantom *A* on left, phantom *B* on right) used in this work. Film positions are shown as dotted lines.

Compensating filters were evaluated using two specially fabricated acrylic step phantoms. Phantom cross section dimensions were 18 cm×13 cm (Fig. 1). The thickness of the phantom was 15 cm. The first phantom (*A*) had a three‐step profile on both sides, whereas the second phantom (*B*) had a three‐step profile on only one side. Each step was 2 cm high and 2 cm deep. Each phantom had a rectangular cavity ($5.5\times 8\;{\text{cm}}^{2}$) that could be filled with bone $\left(\rho =1.65\;\text{gm}/{\text{cm}}^{3}\right)$, cork $\left(\rho =0.3\;\text{gm}/{\text{cm}}^{3}\right)$, or Styrofoam $\left(\rho =0.01\;\text{gm}/{\text{cm}}^{3}\right)$. Cork was used to model lung tissue and the Styrofoam was used to simulate an air cavity. For measurements with a homogeneous phantom, the cavity was filled with acrylic material $\left(\rho =1.18\;\text{gm}/{\text{cm}}^{3}\right)$. Phantom *A* was designed to test compensation for both surface irregularities and internal inhomogeneities. Phantom *B*, on the other hand, allowed for separately studying the effects of surface irregularities and internal inhomogeneities.

The phantoms were irradiated with either a single lateral or parallel‐opposed lateral fields. Co60 (Theratron‐1000, MDS Nordion, Kanata, Ontario, Canada), 6 MV, and 18 MV (Varian 2100C, Varian Corporation, Palo Alto, CA) photon beams were used in this work. Field size was $1610\;{\text{cm}}^{2}$. Treatment plans with and without compensating filters were developed for a homogeneous phantom (cavity filled with acrylic) and for phantoms with different types of inhomogeneities. To evaluate dose compensation in the presence of an asymmetrically located inhomogeneity, the cavity was shifted laterally by 1 cm from the phantom center (Fig. 1). Compensating filters made of refined gypsum were constructed using an automated fabrication system. Refined gypsum with no additives was selected as the filter material because of its relatively low density $\left(\rho =2.0\;\text{gm}/{\text{cm}}^{3}\right)$. This density is large enough to limit filter thickness to 5.5 cm (within accessory mount clearance) and provide compensation for up to 15 cm of missing tissue. At the same time, the density is sufficiently low to make the filter transmission insensitive to common fabrication errors (±1 mm). In our work with gypsum filters, these fabrication errors resulted in <1% transmission error. Similar fabrication errors, if the filters were made of Lipowitz metal alloy (Cerrobend, *p* = 9.4 gm/cm^3^), would have caused 4.4% change in transmission.^9^

The location of the compensation plane was varied as follows: center of phantom, and at ±4 cm laterally from the phantom center (Fig. 1). The beam isocenter was always placed in the compensation plane. This allowed us to evaluate the influence of the location of inhomogeneity on dose compensation when the cavity was located in front of or behind the compensation plane.

Each phantom could accommodate both film and ionization chamber measurements (Fig. 1). Dose distributions were measured in three sagittal planes: right sagittal (RS), center sagittal (CS), and left sagittal (LS). The separation between planes was 4 cm. Radiographic XV2 films (Eastman Kodak Co., Rochester, NY) were used for the measurement of dose distributions. CMS Dynascan (Computerized Medical Systems, Inc., St. Louis, MO) film scanning system was used to scan films. Film optical density was converted to dose using appropriate depth‐corrected H&D curves. The measured dose distributions were compared with FOCUS TPS calculations. An ionization chamber was positioned in the center sagittal plane (CS) and was used for absolute point dose verification. Superposition and Clarkson algorithms were used for compensating filter design and dose calculations.

Finally, inhomogeneity corrected treatment plans with and without compensating filters were developed for several patients with lung tumors. A four‐field (AP/PA and parallel opposed oblique) beam arrangement was used. This beam arrangement was chosen as it is commonly used to produce uniform dose distributions for plans without heterogeneity corrections. The impact of compensating filters on target dose uniformity was evaluated.

## RESULTS

Dose profiles along the central vertical axis in each sagittal plane were evaluated, since the highest dose gradient is expected along this axis. Table I shows the dose variation for a single right lateral beam with no compensating filter and the cavity filled with different materials. As seen in this table, the dose variation ranged from 7.4% to 67% depending on energy, inhomogeneity, and measurement planes. For comparison, in the case of a flat homogeneous phantom with no surface irregularities (left lateral beam incident on phantom *B*), the dose variation for all beam energies was limited to <2%. In general, the variation in dose decreased with increasing photon energy. Except for the bone inhomogeneity, the smallest dose variation was seen for the homogeneous phantom in all planes for all photon energies. For 6 and 18 MV beams, dose variations in the RS and CS planes were similar for bone and homogeneous phantoms. In the LS plane, the dose variation was 2–3% lower for the case of bone filled cavity. In contrast, for the Co60 beam, the lowest dose variation was seen for the homogeneous phantom in all planes.

**Table I:** Dose variation (in percentage) for a right lateral uncompensated beam incident on phantom A.

	Co60			6 MV			18 MV		
Medium	RS	CS	LS	RS	CS	LS	RS	CS	LS
Homo.	13.5	28.0	26.0	11.1	19.5	17.8	7.9	11.4	11.7
Bone	16.4	39.0	30.0	11.0	19.8	14.8	7.4	11.7	9.5
Cork	16.0	46.0	58.0	12.8	30.2	38.2	9.0	14.2	26.8
Air	14.9	46.0	67.0	12.4	31.0	45.2	8.4	14.5	29.7

Dose distributions also showed a significant dependence on the location of the measurement plane. In the planes located beyond the cavity (e.g., LS plane), the dose variation increased with decreasing density of cavity material. In the plane passing through the cavity (CS plane), the dose variation was similar (within 1%) for cork and Styrofoam at all beam energies. In the phantom containing cork or Styrofoam, the dose inhomogeneity was significantly higher than that for the bone phantom. In the planes located upstream of the cavity (e.g., RS plane) the dose variation changed only slightly (≤3%) with the cavity material. Also, for all photon energies and cavity materials, the RS plane dose variation was significantly lower than that in the CS and LS planes.

The introduction of a compensating filter significantly improved dose uniformity in the plane of compensation, however large dose nonuniformities were seen in other planes. Table II shows results obtained for a single compensated beam. The plane of compensation was the CS plane. In this plane, the dose uniformity was greatly improved for all cavity materials. In general, the dose variation was lowest for homogeneous phantom (≤3.5%) and was higher for cork and Styrofoam filled phantoms (up to 6.5%). With the addition of the filter, the dose variation in planes beyond the cavity (LS plane) was significantly reduced for all energies and cavity materials. However, large dose nonuniformities still remained in both RS and LS planes.

**Table II:** Dose variation (in percentage) for a right lateral compensated beam incident on phantom A.

	Co60			6 MV			18 MV		
Medium	RS	CS	LS	RS	CS	LS	RS	CS	LS
Homo.	5.3	2.3	8.4	3.0	3.4	6.5	12.4	0.4	4.2
Bone	14.0	4.5	7.0	5.4	2.8	5.4	10.2	2.3	2.6
Cork	15.0	4.2	19.3	10.6	5.4	16.5	15.4	4.6	15.5
Air	16.7	5.0	23.2	12.2	4.8	18.7	14.0	6.5	20.0

**Figure 2:**
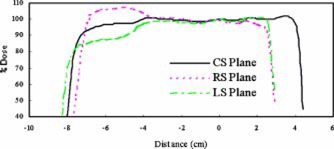
(Color) Dose profiles in the RS, CS, and LS planes for a right lateral 6 MV compensated beam incident on phantom *A* with cork cavity.

Figure 2 compares dose profiles in the three sagittal planes for a single compensated 6MV beam. The cavity material is cork. In addition to providing a uniform dose in the CS plane, the compensator reduced dose variation in the LS plane by more than twice. In the RS plane, however, little change in dose profile was observed.

**Figure 3:**
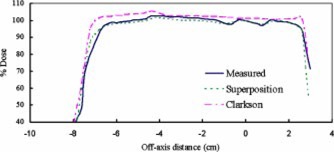
(Color) *A* comparison between measured and calculated dose profiles using Clarkson and superposition algorithms in the RS plane for a pair of compensated parallel‐opposed 6 MV beams incident on phantom *A*.

Figure 3 compares calculated and measured dose profiles in the RS plane for a parallel‐opposed 6 MV compensated beams. Calculations using the superposition algorithm more closely agreed with the measured data than Clarkson calculations. The deviation between measured and calculated profiles was up to 7% for Clarkson algorithm and up to 2% for the superposition algorithm. Furthermore, at points corresponding to cavity interface and abrupt shape changes in the phantom, better than 1% agreement was seen between superposition calculations and the measured profile. Therefore a good overall agreement between calculated (superposition) and measured profile shapes was observed. Similar results (not shown) were obtained for other measurement planes and beam energies. Thus, all calculations reported in this work were performed using the superposition algorithm.

**Figure 4:**
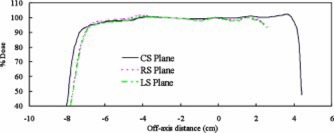
(Color) Calculated dose profiles (superposition algorithm) in the RS, CS, and LS planes for parallel‐opposed compensated 6 MV beam arrangement incident on phantom *A*.

When parallel‐opposed compensated beams were used, the dose variation in all planes decreased to ≤5% for all photon energies and cavity materials Table II(I). Figure 4 illustrates the dose variation in all three sagittal planes for parallel‐opposed compensated 6 MV beams incident on phantom *A* with cork inhomogeneity. The isocenter was located in the CS plane. In this case, the dose was uniform to within 4.5% in all planes.

**Table III:** Dose variation (in percentage) for a pair of parallel‐opposed compensated beams incident on phantom A.

	Co60			6 MV			18 MV		
Medium	RS	CS	LS	RS	CS	LS	RS	CS	LS
Homo.	2.3	2.5	2.3	1.4	2.5	1.4	5.0	0.7	5.0
Bone	4.0	3.0	4.0	3.1	2.2	4.0	3.7	1.4	4.0
Cork	2.0	2.1	2.0	4.2	4.0	3.5	5.0	2.7	5.0
Air	2.0	2.8	2.0	2.0	2.5	4.0	4.3	3.5	5.0

Results obtained for parallel‐opposed lateral beams incident on the asymmetrically shaped phantom *B* filled with cork are shown in Table IV. In this case, quite adequate dose uniformity was obtained with only one compensating filter, provided the compensated beam faced the irregular surface and the plane of compensation was positioned beyond the inhomogeneity. This is illustrated in Table IV (row 5). Here only the right lateral field was compensated and the plane of compensation was the LS plane (Fig. 1). When the superposition algorithm was used, the absolute dose measured in the CS plane with an ionization chamber was in good agreement (within 1.5%) with calculations.

**Table IV:** Dose variation (in percentage) for a pair of parallel‐opposed 6 MV beams incident on phantom B with cork inhomogeneity.

		6 MV		
Beam configuration	Isocenter plane	RS	CS	LS
RLAT with CF; LLAT without CF	CS	4.2	5.3	4.8
Parallel‐opposed compensated	CS	5.2	1.4	4.5
RLAT with CF; LLAT without CF	RS	7.0	11.8	10.6
Parallel‐opposed compensated	RS	1.5	2.5	2.5
RLAT with CF; LLAT without CF	LS	3.9	1.0	2.0
Parallel‐opposed compensated	LS	4.2	1.0	2.1

**Figure 5:**
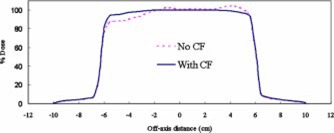
(Color) Target dose profiles (with inhomogeneity corrections) for a lung patient treated with 6 MV four‐field arrangement.

Figure 5 compares target dose profiles for a lung patient planned with and without compensators. All treatment plans included tissue inhomogeneity corrections. When no compensating filters were used, the dose in the target varied from −15% to +5%. The addition of compensating filters resulted in reduction of dose variation to less than 5%.

Based on treatment plans with inhomogeneity corrections, the mean lung dose was underestimated by 6% in the plans without corrections. When compensating filters were added, the mean and maximum lung doses decreased by ~5% and became practically equal to the doses obtained with treatment plans developed assuming the patient to be a homogeneous medium.

## DISCUSSION

Historically, compensating filters have been used as missing tissue compensators to correct for surface irregularities. However, the CT based 3DTPS are also capable of correcting dose distributions for inhomogeneities. Therefore, compensating filters may be designed to compensate for both patient surface irregularities and internal inhomogeneities.

The increased use of compensating filters is facilitated by readily available computer‐driven compensator fabrication systems linked with 3DTPS. In our department, a system consisting of PAR scientific milling machine and FOCUS TPS is being used.

In a 3DTPS, the choice of dose algorithm dictates the accuracy of calculated dose distribution in the presence of tissue inhomogeneities. Compared to Clarkson calculations, the superposition algorithm has been shown to provide better agreement with measured data, especially in the interface region.^19^ This can be explained by the ability of the superposition algorithm to more accurately model changes in scatter radiation due to tissue inhomogeneities and surface irregularities. In the superposition algorithm, which is based on “collapsed cone” dose calculation,^20^ the energy deposition kernels are distorted to account for variations in electron density in each voxel. Our results confirmed that compared to Clarkson algorithm, the superposition algorithm indeed produced better agreement with measured dose profiles. The dose variation reduced from 7% (Clarkson) to <2% (superposition). The agreement between measured and calculated doses was especially improved in the regions corresponding to sharp changes in phantom surface and tissue‐inhomogeneity interface.

The selection of refined gypsum mixture (with no additives) for fabrication of compensating filters was based on its density ($2.0\;\text{gm}/{\text{cm}}^{3}$). This density represents a good compromise between competing requirements for an ideal filter material: the density should be large enough to provide a compensator of manageable dimensions, and yet it should be small enough that common errors in filter fabrication (±1 mm) do not produce measurable errors in dose distribution.

A parallel‐opposed beam configuration is commonly used to compensate for dose variation with depth. However, the presence of surface irregularities and/or tissue inhomogeneities creates dose variations in planes perpendicular to the beam. When a compensating filter is added to the beam, a uniform dose distribution (within 2–3%) is achieved in only one plane (the plane of compensation, CP). Overcompensation (reduced dose) and undercompensation (increased dose) occur in planes above and below the CP respectively. When an inhomogeneity is present, these dose variations are amplified. Indeed, when an inhomogeneity extended beyond the compensation plane (CP), the dose variation in planes beyond CP increased by a factor of 2–3 compared to homogeneous medium. The dose variation was more pronounced at lower photon energies and for low‐density materials (cork and Styrofoam, which represent lung and air filled cavities in patients).

When a pair of compensated parallel‐opposed beams is used, regions of overcompensation due to one beam may overlap with regions of undercompensation from the opposing beam. Thus, the use of compensators in parallel‐opposed beams should produce lateral dose uniformity in addition to depth dose uniformity. Indeed as our measurements have demonstrated, the degree of dose nonuniformity in planes located 4 cm away from CP was reduced from as high as 23% to ~2% when compensators were introduced in Co60 parallel‐opposed beams. Similar effect was observed with 6 MV and 18 MV beams.

There are several publications concerning the use of compensating filters.^9–^, ^13^ However, in most publications, the impact of inhomogeneities was not considered at all. In others, only cork and Styrofoam were studied. In many publications, only one energy (usually 6 MV photons) was considered and no comparison between different algorithms was carried out. Furthermore, dose homogeneity was studied only in the compensation plane. In addition, most of the compensators considered in literature were non‐gypsum based. In our paper, a comprehensive study that considered a large range of inhomogeneities and beam energies was carried out. Also, the ability to provide volumetric dose compensation was evaluated. Our study also compared the accuracy of superposition and Clarkson algorithms in designing compensators. The choice of gypsum material in our work was based on a number of factors, e.g., simple compensator fabrication, reproducible and uniform density filters, cost‐effectiveness (cheapest material available for compensating filter), and suitable density for most clinical applications. However, the relatively low density of gypsum precludes its use in the IMRT

## CONCLUSIONS

Compensating filters may be effectively used for restoring dose uniformity in the presence of surface irregularities and internal inhomogeneities. Computer‐driven systems provide a practical, convenient, and accurate method for compensating filter fabrication. The use of refined gypsum results in manageable size filters that are not sensitive to commonly encountered machining errors. The introduction of compensating filters in parallel‐opposed beams provides lateral dose compensation throughout the target volume. The accuracy of algorithms available in a 3DTPS for designing compensating filters should be experimentally evaluated.
